# Supplementary figures and images for: Dynamic combination of sensory and reward information under time pressure
Source: PLoS Comput Biol. 2018 Mar 27;14(3):e1006070. doi: 10.1371/journal.pcbi.1006070 (PMC5889192; doi:10.1371/journal.pcbi.1006070)

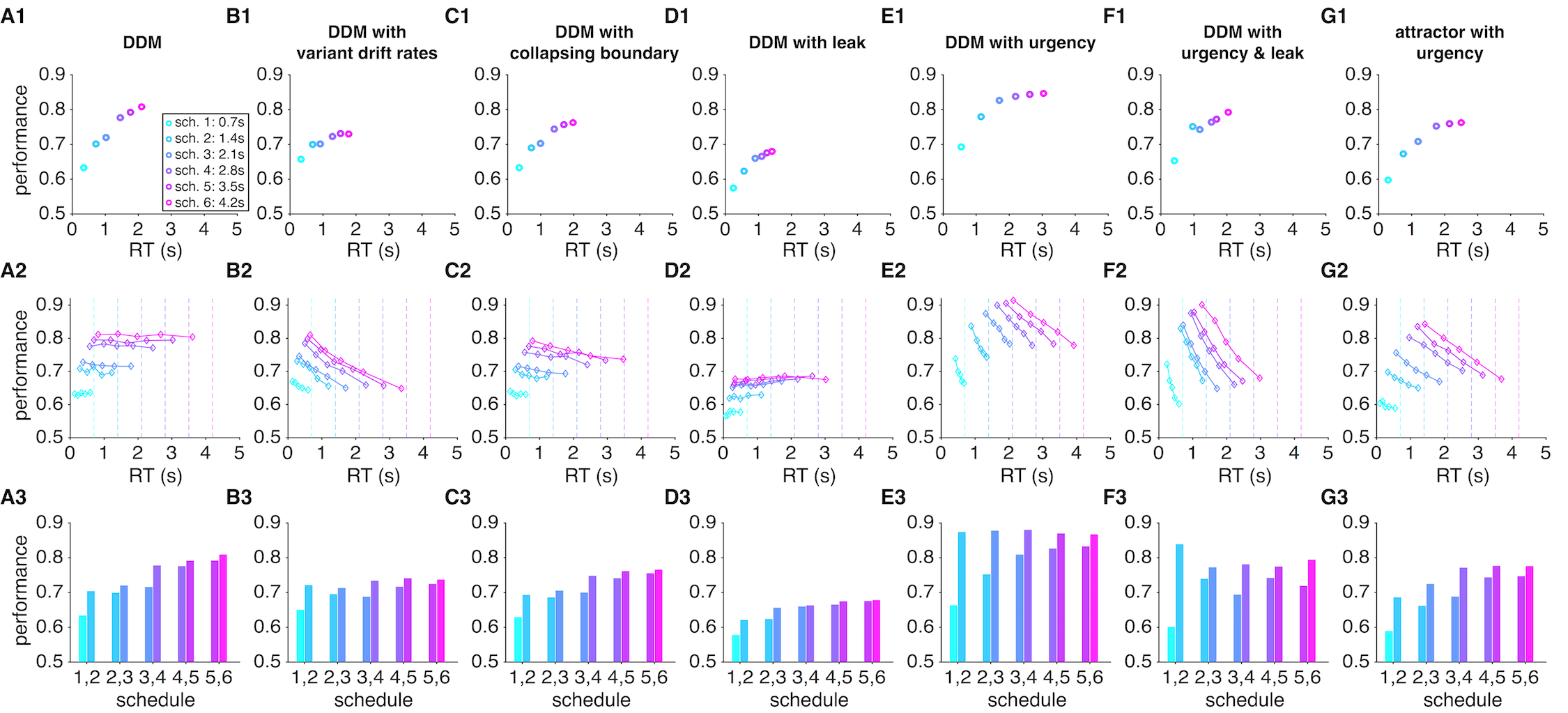

Supplement: S1 Fig — Results for different models are presented on different columns, while panels 1 to 3 of each column plot: the average speed-accuracy tradeoff for a given level of time pressure (panel 1); performance as function of proximity to the deadline (panel 2); and the average performance on RT-matched trials (based on mean) with adjacent levels of time pressure (panel 3). The parameters for each model are selected to show a typical behavior representative of each model. For these simulations, trials in which the decision boundary was not crossed before the deadline were removed from the analysis. (TIFF) [file pcbi.1006070.s001.tiff]
