# Supplementary material for: Dynamic combination of sensory and reward information under time pressure
Source: PLoS Comput Biol. 2018 Mar 27;14(3):e1006070. doi: 10.1371/journal.pcbi.1006070 (PMC5889192; doi:10.1371/journal.pcbi.1006070)
Supplement: S1 Table — Reported are the Akaike information criterion (AIC) values for fitting mean and standard deviation of reaction time and performance over all subjects. The DDM model with urgency and leak provides the best fit between all models. (DOCX) [file pcbi.1006070.s002.docx]

| Model | DDM | DDM with variant drift rates | DDM with collapsing boundary | DDM with leak | DDM with urgency | DDM with urgency & leak | Attractor with urgency |
| --- | --- | --- | --- | --- | --- | --- | --- |
| AIC | 1302.7 | 1589.4 | 1387.4 | 1608.1 | 1216.5 | 1095.8 | 1222.9 |

**Supplementary Table 1**
